# Combining targeted and systematic prostate biopsy improves prostate cancer detection and correlation with the whole mount histopathology in biopsy naïve and previous negative biopsy patients

**DOI:** 10.3389/fsurg.2022.1013389

**Published:** 2022-10-06

**Authors:** Johannes Mischinger, Helmut Schöllnast, Hanna Zurl, Mark Geyer, Katja Fischereder, Gabriel Adelsmayr, Jasminka Igrec, Gerald Fritz, Martina Merdzo-Hörmann, Jörg Elstner, Johannes Schmid, Alfred Triebl, Viktoria Trimmel, Clemens Reiter, Jakob Steiner, Dominik Rosenlechner, Maximilian Seles, Georg P. Pichler, Martin Pichler, Jakob Riedl, Stephanie Schöpfer-Schwab, Jakob Strobl, Georg C. Hutterer, Richard Zigeuner, Karl Pummer, Herbert Augustin, Sascha Ahyai, Sebastian Mannweiler, Michael Fuchsjäger, Emina Talakic

**Affiliations:** ^1^ Department of Urology, Medical University of Graz, Graz, Austria; ^2^ Department of Radiology, Medical University of Graz, Graz, Austria; ^3^ Department of Oncology, Medical University of Graz, Graz, Austria; ^4^ Institute for Pathology, Medical University of Graz, Graz, Austria

**Keywords:** prostate cancer detection, PI-RADS-Version-2, combination of fusion and systematic biopsy, uroNav, biopsy-naïve, previous-negative biopsy, whole mount histopathology

## Abstract

**Objective:**

Guidelines for previous negative biopsy (PNB) cohorts with a suspicion of prostate cancer (PCa) after positive multiparametric (mp) magnetic-resonance-imaging (MRI) often favour the fusion-guided targeted prostate-biopsy (TB) only approach for Prostate Imaging-Reporting and Data System (PI-RADS) ≥3 lesions. However, recommendations lack direct biopsy performance comparison within biopsy naïve (BN) vs. PNB patients and its prognostication of the whole mount pathology report (WMPR), respectively. We suppose, that the combination of TB and concomitant TRUS-systematic biopsy (SB) improves the PCa detection rate of PI-RADS 2, 3, 4 or 5 lesions and the International Society of Urological Pathology (ISUP)-grade predictability of the WMPR in BN- and PNB patients.

**Methods:**

Patients with suspicious mpMRI, elevated prostate-specific-antigen and/or abnormal digital rectal examination were included. All PI-RADS reports were intramurally reviewed for biopsy planning. We compared the PI-RADS score substratified TB, SB or combined approach (TB/SB) associated BN- and PNB-PCa detection rate. Furthermore, we assessed the ISUP-grade variability between biopsy cores and the WMPR.

**Results:**

According to BN (*n* = 499) vs. PNB (*n* = 314) patients, clinically significant (cs) PCa was detected more frequently by the TB/SB approach (62 vs. 43%) than with the TB (54 vs. 34%) or SB (57 vs. 34%) (all *p* < 0.0001) alone. Furthermore, we observed that the TB/SB strategy detects a significantly higher number of csPCa within PI-RADS 3, 4 or 5 reports, both in BN and PNB men. In contrast, applied biopsy techniques were equally effective to detect csPCa within PI-RADS 2 lesions. In case of csPCa diagnosis the TB approach was more often false-negative in PNB patients (BN 11% vs. PNB 19%; *p* = 0.02). The TB/SB technique showed in general significantly less upgrading, whereas a higher agreement was only observed for the total and BN patient cohort.

**Conclusion:**

Despite csPCa is more frequently found in BN patients, the TB/SB method always detected a significantly higher number of csPCa within PI-RADS 3, 4 or 5 reports of our BN and PNB group. The TB/SB strategy predicts the ISUP-grade best in the total and BN cohort and in general shows the lowest upgrading rates, emphasizing its value not only in BN but also PNB patients.

## Introduction

Recently, prostate multiparametric magnetic resonance imaging (mpMRI) and standardized acquisition, interpretation, and reporting of prostate mpMRI guided by the Prostate Imaging Reporting and Data System Version-2 (PI-RADS) score has substantially improved clinically significant (cs) prostate cancer (PCa) diagnosis (1).

Over the last five years an increased csPCa detection rate has been shown with the combination of the targeted-mpMRI/transrectal ultrasound (TRUS)-fusion-guided-prostate-biopsy (TB) approach plus the systematic TRUS biopsy (SB) method in biopsy naïve (BN) only (2–5), BN and previous-negative biopsy (PNB)cohorts (5–8) or mixed groups also including patients with PCa-positive-previous biopsies (PPB) (9–12).

The largest studies (*n* ≥ 1,000) regularly included a high amount of PPB patients and never mentioned the potential bias of an artificially higher PCa prevalence to assess the biopsy test performance in cohorts with previous biopsy (13). In addition, it has been shown that active surveillance, representing PPB patients, was independently associated with an overall and csPCa detection on biopsy (14).

Probably due to historical developments with the introduction of mpMRI and TB of suspicious lesions for patients who were still at risk for PCa after previous negative SB in 2013 (15) and stepwise implementation in BN situations hereafter (2, 3, 16), no direct comparison of TB, SB or TB/SB performances within one study focusing on BN vs. PNB patients seemed relevant, although for both subgroups urologists face different guideline recommendations.

Since the MRI-First study was published in 2019 the recommendation of a combined biopsy approach (TB/SB) for BN patients within the mpMRI pathway (i.e., PI-RADS ≥3) was introduced, by national and international guideline panels (3, 17–20). In contrast, for PNB patients, the European Association of Urology (EAU) advises to perform TB only when prostate mpMRI is positive on the basis of a systematic review and metanalysis, showing that TB had a better accuracy, higher absolute added value for detecting International Society of Urological Pathology (ISUP) grade >2 PCa and less over-detection of insignificant (is) PCa than a classic SB-approach without MRI (20, 21). In this PNB setting the American Urological Association guidelines also state that prebiopsy MRI and MRI targeted biopsy detects more cancers than systematic sampling alone (17, 22) and conclude that mpMRI lesions with very high suspicion for PCa, which were negative on primary TB, should earlier undergo another TB (23, 24).

On the other hand, the Canadian Urological Association suggests to perform TB/SB in PNB situations (18), whereas the European Society of Medical Oncology guidelines generally propose TB with or without SB, when mpMRI is positive (≥PI-RADS 3) (25) but both panels do not explicitly refer to certain publications for this recommendation.

Interestingly, only Preisser and colleagues perceived the investigative flaw of the mpMRI biopsy pathway within PI-RADS 3, 4 and 5 lesions and directly assessed the TB, SB, and TB/SB performance of BN patients compared to PNB patients, resulting in a higher csPCa detection rate with TB/SB in BN patients or men with one-PNB (5). These results would contradict certain international guideline recommendations for PNB patients (17, 20), but the analysis was retrospective, the cohort was small and false negative TB or SB results, as well as the correlation to the whole mount pathology report (WMPR) as reference for the true PCa aggressiveness was not assessed.

The prediction of the WMPR ISUP-grade is highly relevant because untreated localized clinically significant (cs) prostate cancer (PCa) is associated with a higher risk for the development of metastases (26) and PCa-specific death (27).

Last, until now no definitive efforts have been made to distinguish between PI-RADS 2 reports representing target less mpMRI showing csPCa detection rates with SB from 3% to 24% in heterogenous groups (2, 14, 28–30) and visible PI-RADS 2 mpMRI lesions, which have been associated with csPCa in 7% in a multicenter cross-sectional study (31). To verify these PI-RADS 2 lesion results, we also assessed PCa detection rates of in-house PI-RADS 2 lesions after radiologic reevaluation.

In order to improve current evidence, we analyzed the overall and stratified PCa-detection rate by comparing the performance of TB, SB, or TB/SB between BN- and PNB patients with suspicious PI-RADS 2, 3, 4 or 5 lesions. The WMPR of patients who underwent radical prostatectomy (RP) was considered as ISUP-grade reference gold standard.

## Materials and methods

### Patient selection

This retrospective study was approved by the Institutional Review Board (32-091Ex19/20) of the Medical University of Graz (MUG). From January 2018 through August 2020, patients referred to our department to perform fusion biopsy (FB), with an increased prostate specific antigen (PSA) value and/or abnormal digital rectal examination (DRE), suspicious lesions on mpMRI (PI-RADS 2, 3, 4 or 5) and no previous PCa history, were eligible for enrollment. Except for cancer core length, all patient data were reported with respect to the START- Consortium (32).

### Multiparametric MRI

All patients underwent 1,5 or 3 Tesla prostate specific endorectal coil free mpMRI, either in one of 14 extramural Austrian radiologic institutes or intramurally at the department of radiology of the MUG.

In accordance with the international PI-RADS version 2 working group recommendation the imaging protocols of each institution included high resolution T1 (to determine the presence of hemorrhage within the prostate and seminal vesicles) and T2-weighted images in axial, coronal and sagittal plane, diffusion weighted imaging as well as dynamic contrast enhanced weighted images (33). In addition, patients with ≥1 suspicious mpMRI lesion were given a PI-RADS 2–5 to locate and stratify the risk for PCa.

The mpMRI data set and the original PI-RADS reports of all patients were reviewed according to the PI-RADS by 9 radiologic specialists (trained by H.S. and G.F with 7 years of prostate mpMRI-experience each, respectively) but no rerating of in-house mpMRI reports was performed. Subsequently, before prostate biopsy, the outlines of the prostate and suspicious regions that had been identified were labeled in all prostate- and lesion-incorporating axial T2-weighted images by the same reviewing radiologist with the use of the DynaCAD (Philips, USA) software. For data evaluation in cases of multiple lesions, the highest PI-RADS score observed was recorded.

### Prostate biopsy process

Biopsies of suspicious prostatic lesions were performed using the UroNav (2.0, Invivo Corporation-Philips-Gainsville, USA)- TRUS (BK 3000 and E14C4T side-fire probe, Denmark)-fusion guided-prostate biopsy system under oral antibiotic prophylaxis and local anesthesia (15 ml of Lidocaine 1% transrectally injected at the bilateral prostatoseminal angle) by 4 trained and dedicated Urologists (2–5 years of experience) and 4 supervised residents. Either four biopsy cores were obtained in the sagittal plane from one lesion or two biopsy cores from each lesion if ≥2 targets were described. We applied a fan biopsy technique within the largest anticipated diameter of a target lesion (PI-RADS 2, 3, 4 or 5), to represent the target area more precisely.

Hereafter, the UroNav biopsy mode was switched from “target” to “other biopsies” mode (target vanished) and the SB was taken neglecting the former visualized target or performed TB cores. The SB was performed as recommended by the German S-3 guidelines starting within the peripheral zone on the right lobe side first laterally then medially at the base of the prostate performing the same procedure on the contralateral lobe. Hereafter, SB cores were taken from the lateral and medial middle part usually hitting the peripheral, transitional and/or central zone of the prostate.

Last we took at the lateral and medial apex on both sides very steep SBs to clarify potential csPCa in the anterior peripheral zone and/or fibromuscular stroma. All cores were separately documented and collected (19).

Finally, biopsy cores and in-house RP specimens were analyzed by a uro-pathologic working-group comprising nine specialists of the MUG under supervision of S.M. (18 years of uro-pathologic experience) with a thorough reassessment of equivocal histology results. In case of a PCa diagnosis, the highest ISUP-grade of each biopsy method or the WMPR was recorded (34). According to the prostate biopsy result corresponding EAU risk groups for biochemical recurrence of localised and locally advanced PCa was assessed. The EAU intermediate (PSA 10–20 ng/ml or ISUP 2/3 or cT2b) and high risk (PSA >20 ng/ml or ISUP 4/5 or ≥cT2c) groups were defined as csPCa (20, 35).

### Statistical analysis

The chi-squared or Fisher's exact test were used as appropriate to evaluate proportions' differences of two or more nominal-scaled variables. McNemar's test was applied to match paired nominal data (biopsy-method and WMPR with respect to BN- or PNB-status). A two-sided *p* < 0.05 was considered statistically significant. All statistical analyses were performed with the JMP 15.0.0 software (SAS Institute Inc., USA).

## Results

### Study population

**Figure 1:**
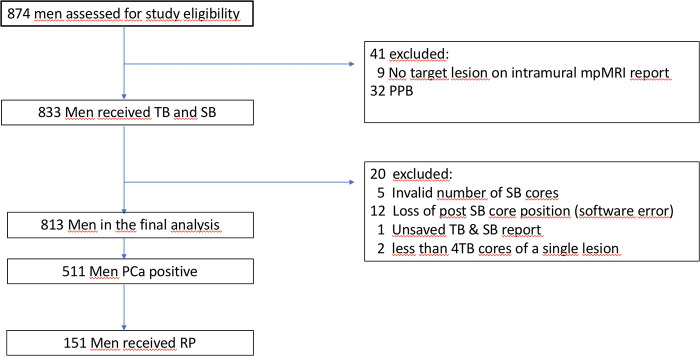
Flowchart describing the study sequence of men who received multiparametric (mp) magnetic resonance imaging (MRI) due to elevated prostate specific antigen and/or suspicious digital rectal examination. Target Biopsy (TB), Systematic Biopsy (SB), Prostate Cancer (PCa), PCa Positive Previous Biopsy (PPB), Radical Prostatectomy (RP).

In total, 874 patients were referred for FB, whereas 61 patients were excluded due to a missing target lesion, PPB situation or inappropriate biopsy procedure (Figure 1).

Finally, of 813 men who participated in this study, 511 (63%) showed a PCa positive biopsy and 151 (30%) underwent RP (87% of these were performed intramurally). Baseline patient characteristics with additional respect to csPCa and biopsy-history are shown in Table 1.

**Table 1:** Characteristics of the study cohort with respect to clinically significant (cs) prostate cancer (PCa) diagnosis (defined by the “European Association of Urologists risk stratification”) and the biopsy history.

Characteristics	Total	csPCa	No csPCa	*p*	BN	PNB	*p*
Age, median (IQR)	67 (61–73)	69 (62–75)	65 (58–71)	**<0**.**0001**	66 (60–73)	67 (61–73)	0.1
Type of mpMRI, 3 vs. 1.5 Tesla (%)	90 vs. 10	90 vs. 10	88 vs. 12	0.1	90 vs. 10	90 vs. 10	0.5
Days from mpMRI to FB, median (IQR)	83 (58–111)	77 (50–104)	87 (65–125)	**<0**.**0001**	78 (49–105)	90 (69–128)	**<0**.**0001**
No. of extramural/intramural mpMRIs (%)	97/3	98/2	97/3	0.3	97/3	98/2	0.2
PSA, median (IQR)	6.8 (4.9–9)	7.2 (5–10.7)	6.3 (4.6–8.5)	**<0**.**0001**	6 (4.4–9)	7 (5.4–10.4)	0.3
PSA-density, median (IQR)	0,14 (0.09–0.22)	0.16 (0.11–0.27)	0.11 (0.08–0.17)	**<0**.**0001**	0,14 (0.09–0.22)	0,14 (0.09–0.24)	0.9
Clinical Tumor (cT) DRE result, *n* (%)							
1c	572 (70)	272 (61)	300 (82)	**<0**.**0001**	333 (67)	239 (76)	**0**.**005**
2a	86 (11)	52 (12)	34 (9)	0.3	57 (11)	29 (9.2)	0.4
2b	97 (12)	74 (17)	23 (6)	**<0**.**0001**	64 (13)	33 (10.5)	0.3
2c	53 (6)	44 (10)	9 (2)	**<0**.**0001**	41 (8)	12 (4)	**0**.**01**
3	5 (1)	3 (0.6)	2 (0.5)	0.8	4 (1)	1 (0.3)	0.4
UroNav Fusion Prostate volume, ml, median, (IQR)	46 (34–64)	42 (31–58)	52 (38–74)	**<0**.**0001**	43 (32–60)	51 (37–71)	**<0**.**0001**
mpMRI Dynacad Target volume, ml, median (IQR)	0.81 (0.45–1.45)	0.87 (0.46–1.66)	0.74 (0.44–1.3)	**0**.**001**	0.78 (0.45–1.43)	0.82 (0.5–1.48)	0.2
No. of visible mpMRI-lesions, median (IQR, min.-max.)	1 (1-1, 1–3)	1 (1-1, 1–3)	1 (1-1, 1–3)	0.1	1 (1-1, 1–3)	1 (1-1, 1–3)	0.3
Cores taken per target lesion, median (IQR, min.-max.)	4 (4-4, 4–8)	4 (4-4, 4–8)	4 (4-4, 4–6)	0.06	4 (4-4, 4–6)	4 (4-4, 4–8)	0.5
Cores taken by systematic biopsy, median, (IQR, min.-max.)	12 (12-12, 12-12)	12 (12-12, 12-12)	12 (12-12, 12-12)	1	12 (12-12, 12-12)	12 (12-12, 12-12)	1
Number of RPs, *n* (%)	151 (100)	148 (98)	3 (2)	**<0**.**0001**	110 (22)	41 (13)	**0**.**002**
RP performed intramural/extramural, *n* (%)	131 (87)/20 (13)	128 (86)/20 (14)	3 (100)/0	**<0.0001/-**	97 (88)/13 (12)	34 (83)/7 (17)	0.4
Months to RP, median (IQR)	4 (3–4)	4 (3–4)	5 (3–6)	0.7	4 (3–4.5)	4 (3–4)	0.5

Fusion biopsy (FB), Biopsy-naïve (BN = green box), previous-negative biopsy (PNB = grey box) patients, multiparametric (mp) Magnetic Resonance Imaging (MRI), Prostate Specific Antigen (PSA), Radical Prostatectomy (RP).

Bold *p*-values indicate clinical significance.

### Assessement of PCa-detection according to the Pi-RADS, biopsy mode and biopsy-history

The combined approach of TB/SB detected significantly more csPCa than the TB- or SB-approach alone (Table 2). This finding applied to the overall (55% vs. 46% or 48%; both: *p* < 0.0001), BN- (62% vs. 54% or 57%; both:*p* < 0.0001) or PNB-cohort (43% vs. 34% or 34%; both: *p* < 0.0001). According to PI-RADS 3, 4 or 5 lesions, the highest rate for csPCa diagnosis was also observed by TB/SB in the total cohort, BN- or PNB-group, respectively. In contrast, we observed no statistically significant difference for the detection of csPCa within PI-RADS 2 lesions.

**Table 2:** Biopsy performance of combined biopsy (TB/SB), target biopsy (TB) and systematic biopsy (SB) in association with overall / clinically significant (cs) prostate cancer (PCa) according to the “Prostate Imaging Reporting and Data System Version 2 (PI-RADS) 2–5 report and biopsy-naïve (BN = green box)- and previous-negative biopsy (PNB = grey box) patients. Cs- or insignificant (is)- PCa was defined according to the “European Association of Urologists” risk groups with respect to the International Society of Urological Pathology (ISUP) grading.

Specimen	TB	SB	*p*	TB/SB Biopsy	*p* (TB/SB vs.TB)/*p* (TB/SB vs.SB)
overall PCa, *n* (%)	421 (52)	459 (57)	**0**.**001**	511 (63)	**<0.0001/<0.0001**
cs PCa, *n* (%)	375 (46)	391 (48)	0.2	445 (55)	**<0.0001/<0.0001**
csPCa PI-RADS 2, *n* (%)	2 (5)	1 (3)	0.6	3 (8)	0.3/0.2
csPCa PI-RADS 3, *n* (%)	37 (18)	51 (25)	**0**.**02**	61 (30)	**<0.0001/0.002**
csPCa PI-RADS 4, *n* (%)	185 (51)	191 (52)	0.5	222 (61)	**<0.0001/<0.0001**
csPCa PI-RADS 5, *n* (%)	151 (71)	148 (70)	0.5	159 (75)	**0.005**/**0.001**
cs PCa solely diagnosed by one biopsy method, *n* (%)	43 (10)	60 (13)	0.09		** **
BN cs PCa, *n* (%)	268 (54)	283 (57)	0.1	310 (62)	**<0.0001/<0.0001**
BN cs PCa PI-RADS 2, *n* (%)	1 (9)	0	0	1 (9)	1/0
BN cs PCa PI-RADS 3, *n* (%)	23 (21)	32 (29)	**0**.**049**	38 (34)	**0.0001**/**0.01**
BN cs PCa PI-RADS 4, *n* (%)	132 (56)	141 (60)	0.1	155 (66)	**<0.0001/0.0002**
BN cs PCa PI-RADS 5, *n* (%)	112 (80)	110 (79)	0.5	116 (83)	**0.0455**/**0.01**
PNB cs PCa, *n* (%)	107 (34)	108 (34)	0.9	135 (43)	**<0.0001/<0.0001**
PNB cs PCa PI-RADS 2, *n* (%)	1 (4)	1 (4)	1	2 (8)	0.3/0.3
PNB cs PCa PI-RADS 3, *n* (%)	14 (16)	19 (21)	0.2	23 (26)	**0.003**/**0.0455**
PNB cs PCa PI-RADS 4, *n* (%)	53 (41)	50 (39)	0.6	67 (52)	**0.0002/<0.0001**
PNB cs PCa PI-RADS 5, *n* (%)	39 (54)	38 (53)	0.7	43 (60)	**0.0455**/**0.03**
Is PCa, *n* (%)	47 (6)	68 (8)	**0**.**007**	66 (8)	**0.003**/0.67
BN Is PCa, *n* (%)	37 (7)	47 (9)	0.1	46 (9)	0.1/0.8
PNB Is PCa, *n* (%)	10 (3)	21 (7)	**0**.**02**	20 (6)	**0.01**/0.7

Bold *p*-values indicate clinical significance.

The stratified biopsy PCa detection rate is presented in Table 3. BN-TB/SB and PNB-TB/SB detected csPCa in 62% and 43% (*p* < 0.0001), respectively. In addition, the TB- (BN 54% and PNB 34%; *p* < 0.0001) or SB-approach (BN 57% and PNB 34%; *p* < 0.0001) detected more csPCa in BN-situations.

**Table 3:** Biopsy naïve (BN = green box) and previous-negative-biopsy (PNB = grey box) patients and relations to the biopsy technique (combined biopsy (TB/SB), target biopsy (TB) and systematic biopsy (SB) and “Prostate Imaging–Reporting and Data System Version 2 (PI-RADS) 2–5 evaluating radiologist report. Clinically significant (cs) prostate cancer (PCa) or insignificant (is) PCa was defined according to the “European Association of Urologists” risk groups.

Characteristics	BN	PNB	*p*
Total, n (%; median, IQR, min.-max.)	499 (61)	314 (39; 1, 1–2, 1–6)	** **
PI-RADS 2, *n* (%)	11 (2)	25 (8)	**0**.**0001**
PI-RADS 3, *n* (%)	112 (22)	89 (28)	0.06
PI-RADS 4, *n* (%)	236 (47)	128 (42)	0.07
PI-RADS 5, *n* (%)	140 (28)	72 (23)	0.1
csPCa detection (TB/SB) PI-RADS 2, *n* (%)	1 (9)	2 (8)	0.9
csPCa detection (TB/SB) PI-RADS 3, *n* (%)	38 (34)	23 (26)	0.2
csPCa detection (TB/SB) PI-RADS 4, *n* (%)	155 (66)	67 (52)	**0**.**01**
csPCa detection (TB/SB) PI-RADS 5, *n* (%)	116 (83)	43 (60)	**0**.**0002**
overall PCa detection TB/SB, *n* (%)	356 (71)	155 (49)	**<0**.**0001**
cs PCa detection TB/SB, *n* (%)	310 (62)	135 (43)	**<0**.**0001**
cs PCa detection TB, *n* (%)	268 (54)	107 (34)	**<0**.**0001**
cs PCa detection SB, *n* (%)	283 (57)	108 (34)	**<0**.**0001**
cs PCa detection solely by TB total cohort, *n* (%)	21 (4)	22 (7)	0.08
cs PCa detection solely by TB csPCa-cohort, *n* (%)	21 (7)	22 (16)	**0**.**002**
cs PCa detection solely by SB total cohort, *n* (%)	34 (7)	26 (8)	0.44
cs PCa detection solely by SB csPCa-cohort, *n* (%)	34 (11)	26 (19)	**0**.**02**
cs PCa detection simultaneously by TB/SB, *n* (%)	255 (82)	87 (64)	**<0**.**0001**
Is PCa detection TB/SB, *n* (%)	46 (9)	20 (6)	0.1
Is PCa detection TB, *n* (%)	37 (7)	10 (3)	**0**.**01**
Is PCa detection SB, *n* (%)	47 (9)	21 (7)	0.2
Whole mount pathology ISUP score after RP			
ISUP 1, *n* (%)	6 (5)	2 (5)	0.8
ISUP 2, *n* (%)	39 (36)	11 (27)	0.3
ISUP 3, *n* (%)	42 (38)	15 (36)	0.9
ISUP 4, *n* (%)	9 (8)	7 (17)	0.1
ISUP 5, *n* (%)	14 (13)	6 (15)	0.8

International Society of Urological Pathology (ISUP), Radical prostatectomy (RP).

Bold *p*-values indicate clinical significance.

Irrespective of the biopsy technique, the highest amount of csPCa in the total cohort, BN- and PNB-group was found by decreasing order in PI-RADS 5, 4, 3, and 2 scores, respectively (Table 2). BN-patients were associated with more csPCa of PI-RADS 4–5 lesions after TB/SB (0.01 and 0.002) was performed compared to the PNB cohort but this difference could not be found for PI-RADS 2 or 3 reports.

In case of csPCa-diagnosis, the SB-mode (BN 7% and PNB 16%; *p* = 0.002), as well as the TB-approach (BN 11% and PNB 19%; *p* = 0.02) missed csPCa more often in PNB-patients. In addition, isPCa was significantly more often detected by TB in BN- (7%) than in PNB- (3%) patients (*p* = 0.01). Furthermore, within the group of PNB-patients, TB detected isPCa less frequently than did SB (3% vs. 7%, *p* = 0.02) or TB/SB (3% vs. 6%, *p* = 0.01). No statistically significant difference according to biopsy mode for the detection of isPCa in BN patients (TB/SB 9% vs. TB 7%; *p* = 0.1 or SB 9%; *p* = 0.8) was recorded.

### Diagnostic accuracy of the prostate biopsy method (ISUP-grade) on the basis of the WMPR and with respect to biopsy history

The comparison of BN vs. PNB patients regarding each biopsy technique differed only for the ISUP-grade agreement (47 vs. 27%; *p* = .0.02) and upgrading (37 vs. 66%; *p* = 0.002) of the SB approach, respectively.

The TB/SB ISUP-grade showed a significantly higher agreement with the WMPR ISUP-grade than the TB or SB method (both *p* = 0.001; Table 4). Comparable agreement results were equally observed for BN-patients (BN-TB/SB 55% vs. BN-TB 41%; *p* = 0.003 or -BN-SB 47%; *p* = 0.01) and in the PNB setting for PNB-TB/SB (46%) vs. PNB-SB (27%; *p* = .0.03) patients, but there was no difference between PNB-TB/SB (46%) vs. PNB-TB (39%; *p* = 0.2).

**Table 4:** Accurracy of Target Biopsy (TB), Systematic Biopsy (SB) and combined biopsy (TB/SB) to predict the International Society of Uropathological Pathology (ISUP) score of the whole-mount pathology report (WMPR) of patients who consented radical prostatectomy (*n* = 151). Agreement = dark green, upgrading = red, downgrading = yellow. Not applicable (n.a.).

	Total TB/SB	BN TB/SB	PNB TB/SB	*p* BN vs. PNB TB/SB	Total TB	BN TB	PNB TB	*p* BN vs. PNB TB	Total SB	BN SB	PNB SB	*p* BN vs. PNB SB	*p* Total TB/SB vs.TB/SB / TB vs.SB	*p* BN TB/SB vs.TB/SB / TB vs.SB	*p* PNB TB/SB vs.TB/SB / TB vs.SB
ISUP Agreement with **WMPR**, *n* (%)	80 (53)	61 (55)	19 (46)	0.3	61 (40)	45 (41)	16 (39)	0.8	63 (42)	52 (47)	11 (27)	**0**.**02**	**0.001/ 0.001 /** 0.8	**0.003/0.01 /** 0.3	0.2**/0.03 /** 0.3
ISUP Upgrading with **WMPR,** *n* (%)	43 (28)	28 (25)	15 (37)	0.2	75 (50)	55 (50)	20 (49)	0.9	68 (45)	41 (37)	27 (66)	**0**.**002**	**<0.0001/<0.0001/** 0.4	**<0.0001/0.0003/ 0.02**	**0.03/0.0005 /** 0.09
Upgrading ISUP 1 to ISUP ≥2 by **WMPR,** *n* (%)	14 (9)	10 (9)	4 (10)	0.9	19 (13)	15 (14)	4 (10)	0.5	20 (13)	14 (13)	6 (15)	0.8	0.09/**0.03** / 0.8	0.1/**0.03** / 0.05	1/0.3 / 0.3
Upgrading ISUP 2 to ISUP ≥3 by **WMPR,** *n* (%)	20 (13)	13 (12)	7 (17)	0.4	26 (17)	18 (16)	8 (20)	0.6	22 (15)	14 (13)	8 (20)	0.3	**0.03/**0.5 / 0.3	**0.03/**0.7 / 0.2	0.6/0.7 / 1
ISUP Downgrading with **WMPR,** *n* (%)	28 (19)	21 (19)	7 (17)	0.8	15 (10)	10 (9)	5 (12)	0.6	20 (13)	17 (15)	3 (7)	0.2	**0.003/0.005** / 0.3	**0.001/0.045** / 0.1	0.2/**0.045** / 0.4
Downgrading to ISUP 2 by **WMPR,** *n* (%)	14 (9)	11 (10)	3 (7)	0.6	6 (4)	5 (4)	1 (2)	0.6	11 (7)	9 (8)	2 (5)	0.5	**0.005**/0.08 / 0.1	**0.01/**0.2 / 0.2	0.2/0.3 / 0.6
Downgrading to ISUP 1 by **WMPR,** *n* (%)	2 (1)	2 (2)	0	0.4	0	0	0	-	2 (1)	2 (2)	0	0.4	n.a./1 / n.a.	n.a./1 / n.a.	n.a./n.a. / n.a.

Biopsy Naïve (BN)- or Previous-Negative Biopsy (PNB)-cohort.

Bold *p*-values indicate clinical significance.

Furthermore, in total (TB/SB 28% vs. TB 50% and SB 45%; both *p* < 0.0001) and according to BN- (BN-TB/SB 25% vs. BN-TB 50% and BN-SB 37%; *p* < 0.0001 and *p* = 0.0003) and PNB- (PNB-TB/SB 37% vs. PNB-TB 49% and PNB-SB 66%; *p* = 0.03 and *p* = 0.0005) patients, TB/SB was statistically significantly less upgraded by the WMPR than the TB or SB.

Ultimately, ISUP downgrading with the WMPR was significantly more frequently observed with TB/SB (19%) than with TB (10%; *p* = 0.003) or SB (13%, *p* = 0.005). We observed comparable results for BN patients and PNB-TB/SB (17%) vs. PNB-SB (7%; *p* = 0.045), but no difference with respect to PNB-TB.

## Discussion

In this study we observed that the TB and SB approaches seem to be equally effective in detecting csPCa in the overall patient cohort, BN and PNB patients with exception for BN-PI-RADS 3 lesions. The currently published literature offers conflicting data on this topic with a meta-analysis on the one hand, showing that BN patients who underwent mpMRI informed TB plus/minus SB were more likely to be diagnosed with csPCa compared to those who underwent SB alone (36), whereas more than a half of included studies did not perform TB and SB within one patient and therefore hamper conclusions. On the other hand and in line with our own results, another recent meta-analysis (37) and a prospective multicenter study (3) were equally not able to demonstrate a significant difference regarding the detection rate of csPCa between TB and SB in BN-men.

These results may substantiate the hypothesis that mpMRI informed biopsy strategies might improve concomitant SB results compared to classic single SB, maybe due to post TB needle tracks making SB more likely to puncture sample tissues of the same position as TB, even if the performing surgeons are blinded (38, 39).

Preisser et al. reported an identical biopsy sequence and SB procedure compared to our study and also found comparable TB and SB csPCa detection rates in the total (45% vs. 46%)-, BN (52% vs. 54%)- and PNB (32% vs. 31%)-patient cohort, respectively (5).

A systematic review by Drost and colleagues (21) and a prospective study of Exterkate et al. (40) directly compared the mpMRI-pathway against SB in mixed patient cohorts but excluded PPB patients. They favored the TB approach for the detection of csPCa, but lacked a general comparison with TB/SB or WMP as reference for definitive implications.

Therefore, nowadays the classic SB approach precluding the mpMRI pathway has lost validity. Some authors conclude that the additional value of performing SB after TB is limited in patients with PI-RADS 5 lesions with or without a PSA density (PSAD) ≥0.15 or anterior lesions and thus could be omitted (41). In contrast, others proclaim that they failed to identify those patients who might safely benefit from TB alone and recommend SB since TB/SB have a higher detection rate of csPC (8, 42) and false negative TB missing relevant PCa in 9%–27% of heterogenous cohorts (7, 10, 11, 42). Nevertheless, it was not within the focus of this work to assess the influence of a certain additional PSAD cut-off or specific tumor location on the csPCa detection rate of patients.

However, according to PI-RADS 3, 4 or 5 in BN-men of our cohort, the TB/SB approach found significantly more csPCa than TB or SB alone, and thereby confirms recent studies that analyzed BN patients with a suspicious PI-RADS 3, 4 or 5 lesion (2, 4, 5, 7). In addition, and in line with Preisser et al. (5) and Hofbauer et al. (7) who showed the highest absolute numbers for csPCa diagnosis in BN and PNB patients for the TB/SB approach vs. TB or SB (no *p*-value), we were also able to demonstrate a significantly improved PI-RADS 3, 4 or 5 csPCa-detection rate for the TB/SB strategy in our PNB patient cohort. The higher csPCa detection rate in BN patients was independent of the biopsy strategy but seemed to be triggered only by PI-RADS 4 and 5 lesions after TB/SB.

PI-RADS 2 targets of our total cohort were associated with csPCa in 8% after TB/SB.

In this context, within a large and multi-institutional PCa-focus panel, 614 PI-RADS 2 lesions received predominantly TB/SB and were related with ISUP ≥2 PC in 7% (31). Moreover, despite PI-RADS 2 lesions mainly affected PNB-men, the csPCa-detection rate of PI-RADS 2 was equal between TB, SB, and TB/SB with respect to the total cohort, BN or PNB patients. Due to the low number, these PI-RADS 2 results must be interpreted with great caution, even if they suppose that a TB might be sufficient for patients who wish to clarify the low csPCa risk in reevaluated PI-RADS 2 lesions.

In our PNB-cohort we observed that isPCa occurred less frequently in TB than SB (3% vs. 7%, *p* = 0.02) or TB/SB (3% vs. 6%, *p* = 0.01). This finding is in line with larger cohorts which mainly included rebiopsy and PPB patients (9, 10, 12) but also PNB patients showing less isPCa (ISUP-1) with TB (6%) than SB (9%) or TB/SB (12%; no *p*-values) (5). Interestingly, we did not observe a significant difference regarding the detection of isPCa according to the biopsy method in BN men (TB 7% vs. SB 9% vs. TB/SB 9%), probably due to a higher isPCa detection rate after BN-TB (7%) than PNB-TB (3%; *p* = 0.01). This might lead to the assumption that in BN-situations the positive csPCa detection effect of TB/SB is not foiled by an overdetection of isPCa due to the SB-method. Corroborating our results, several other authors also described matchable isPCa detection rates between TB and SB, or TB/SB in BN patients with PI-RADS 3, 4 or 5 lesions, respectively (2, 4, 5, 7, 37).

Anyhow, we want to highlight the important finding that within csPCa-positive patients, a false negative TB-approach was significantly more often observed in PNB- than in BN-patients (19% vs. 11%; *p* = 0.02).

In this context it has to be emphasized that considering the guideline recommendation to solely perform TB after previous negative biopsy in patients with PI-RADS ≥3 reports, csPCa would have been missed in only 4% of PNB-patients with PI-RADS 2 lesions, but in 19% of csPCa-patients of the PNB-cohort.

In our overall patient cohort, we observed that 30% of PCa patients received a RP, which seems to represent a realistic threshold for surgical treatment decisions, assumed that the FB study cohort is larger (31%–39%) (2, 10, 12, 42). Interestingly, TB/SB predicts the WMPR-ISUP grade in our total- and BN-cohort significantly better than the TB or SB approach alone. These agreement percentage relations are in concordance with results from heterogenous biopsy cohorts by for example Diamand et al. (6% PPB patients) (43) (TB/SB 63% vs. TB 51% vs. SB 49%; *p* < 0.001), as well as Ploussard et al. (no PPB group) (44) (TB/SB 52% vs. TB 45% vs. SB 36%; *p* < 0.001), or a BN patient cohort by Maxeiner et al. (TB/SB 33% vs. TB 27% vs. SB 29%; no *p*-value) (4).

More importantly, we found within the total-, BN and PNB-cohorts significantly less upgrading of TB/SB-ISUP results by the WMPR than by either biopsy technique alone. Our upgrading results are substantiated by Ahdoot et al. who included BN- and previous biopsy (PPB fraction of 48%) patients (TB/SB 14% vs. TB 31% vs. SB 42%), Diamand et al. (TB/SB 24% vs. TB 39% vs. SB 43%), Ploussard et al. (TB/SB 32% vs. TB 41% vs. SB 57%), and a BN group described by Maxeiner et al. (TB/SB 16% vs. TB 34% vs. SB 29%). Nevertheless, higher downgrading rates for TB/SB in the total- and BN cohort seem to represent a fact that has to be accepted for an improved agreement and lower upgrading rates (10, 43, 44). Anyhow, downgrading to ISUP-1 PCa is scarce, which is in concordance with our own data (total = 1%, BN = 2%) and the data of Ahdoot et al. (3.7%) (10). Hence, we do believe that the TB/SB approach might decrease the diagnostic uncertainty in BN- and PNB-patients and thus may reduce over-, as well as undertreatment after PCa diagnosis.

To the best of our knowledge, this is the first study to investigate the PCa-detection rate of PPB-free-patients with a suspicious PI-RADS 2, 3, 4, or 5 lesion plus a direct comparison of BN- and PNB-patients regarding the biopsy-modality outcomes and WMPR as a reference test in patients who chose RP as their curative treatment in a large tertiary academic single center setting. Furthermore, we believe that selecting EAU risk groups as level for csPCa is more appropriate, as they essentially base on the D'Amico's classification system, which assessed the risk of biochemical recurrence in patients, who received curative treatment because of localised and locally advanced PCa (35).

Nevertheless, our study is not devoid of limitations: Performing SB always after TB seems to be common practice also in other analysis (4, 5, 7, 42) and may be criticized as it could lead to a bias arising from the knowledge of just previously performed TB. On the contrary performing SB first could impair the diagnostic performance of the TB by SB induced bleeding artifacts (3). Furthermore, SB has been shown to lead to more 30-day-complications than TB alone, although serious adverse events were comparable (SB 2% vs. TB 1.6%) (16) and in general severe complications after FB are rare (45).

In our perioperative FB setting all patients were informed about possible complications and encouraged to visit our outpatient clinic in case of emergency.

We mainly found Grade II (*n* = 33) or IIIa (*n* = 13) complications due to antibiotic therapy or catheterization, respectively. In contrast, Grade I (*n* = 5), IIIb (*n* = 1) or IVa (*n* = 1) complications were seldom, whereas the latter were associated with endoscopic clipping of a rectal bleeding and intensive care monitoring after pulmonary embolism and deep vein thrombosis.

In total, we observed one idiopathic post biopsy fall and conservatively treated fracture of the bridge of nose, gross hematuria (*n* = 7), acute urinary retention (*n* = 8), urinary infection (*n* = 28), rectal bleeding (*n* = 2) and fever or shivering with or without bacteremia (*n* = 8) but no difference according to biopsy history was found.

Due to the retrospective complication rate data acquisition, we could not account for patients who were not bothered about clinically minor or self-limiting symptoms (i.e., hematuria or hematospermia). Probably therefore, most complications stratified by Clavien-Dindo (46) in our study were Grade ≥ II (*n* = 48; 6%) but seemed to be within the 2%–10% range of TRUS biopsy studies reporting on ≥Grade II complications (45, 47, 48).

Furthermore, the clinical parameters which lead to the mpMRI, were gathered retrospectively after referral for FB. Anyhow, intramural PI-RADS reevaluation, biopsy results, intraoperative DRE and RP outcome were gathered prospectively.

Unfortunately, we were not able to assess all treatment decisions after PCa diagnosis owing to patients lost to follow-up. Despite mainly features of a single focus lesion were evaluated, we only accounted for the highest PI-RADS score if more than 1 target was present. In addition, we could not provide the PI-RADS intra and interclass correlation coefficients because none of the inhouse radiologists reevaluated the same mpMRI report again or regularly rerated a patients' mpMRI data set of inhouse colleagues.

The small number of inhouse PI-RADS 2 lesions may confine the generalizability of our results but is explainable by a broad agreement to not classify PI-RADS 2 reports as suspicious. As a result, these patients are referred only sporadically for invasive diagnostic procedures, despite upgrading of PI-RADS 2 reports has been shown to be nearly 18% (30). In addition, 89% of inhouse PI-RADS 2 results represented downgraded extramural PI-RADS ≥3 reports (data not shown), probably relying on a recently attested moderate interobserver agreement for PI-RADS version 2 (49).

Last, the missing additional centralized review of pathology reports by one pathologist may be interpreted as a drawback. However, all biopsy cores were intramurally sampled and hereafter like in-house WMP specimens assessed by our uro-pathologic specialists (from one of Europe's largest pathologic institutes) with respect to the latest ISUP-scoring (34). This workflow enables highly accurate and reproducible biopsy core results and a higher concordance with the final pathology (50).

## Conclusions

In conclusion, the TB/SB strategy seems to detect a significantly higher number of csPCa within PI-RADS 3, 4 or 5 reports, both in BN and PNB men. Moreover, the TB-, SB- and TB/SB-approaches seem to be equivalent to clarify reviewed PI-RADS 2 lesions. In the group of csPCa-positive-patients, the avoidance of the SB would have missed more csPCa in PNB-men. The TB/SB technique predicted the ISUP-grade best in the total- and BN patient cohorts and in general showed the lowest upgrading rates. Hence, the TB/SB workflow should also be recommended for PNB patients.

## Contribution to the field statement

Fusion-guided targeted prostate-biopsy (TB) and concomitant TRUS-systematic biopsy (SB) are recommended for biopsy naïve (BN) patients with a suspicion of prostate cancer (PCa) after positive multiparametric (mp) magnetic-resonance-imaging (MRI).

In contrast, guidelines for previous negative biopsy (PNB) cohorts are inconsistent and often favour the TB only approach, lacking Prostate Imaging Reporting and Data System (PI-RADS) 2 lesions, biopsy approach comparison within BN vs. PNB patients and its prediction of the whole mount pathology report (WMPR).

This is the first study to investigate the PCa-detection rate of patients with a suspicious PI-RADS 2, 3, 4 or 5 lesion plus the direct comparison of BN- and PNB-patients regarding the biopsy-modality outcomes and International Society of Urological Pathology (ISUP)-grade of the WMPR as a reference test.

We observed, that the combined approach (TB/SB) detects a significantly higher number of clinically significant (cs) PCas within PI-RADS 3–5 reports, both in BN and PNB men, whereas TB sufficiently clarifies reviewed PI-RADS 2 lesions. In the group of csPCa-positive-patients, the avoidance of the SB would have missed more csPCa in PNB-men. Furthermore, the TB/SB technique predicts the ISUP-grade best in the total and BN patient cohort and in general showed the lowest upgrading rates, emphasizing its need for recommendation in BN and PNB patients.

## Data Availability

The raw data supporting the conclusions of this article will be made available by the authors, without undue reservation.
